# Fast versus slow infusion of 20% albumin: a randomized controlled cross-over trial in volunteers

**DOI:** 10.1186/s40635-022-00458-3

**Published:** 2022-07-18

**Authors:** Markus Zdolsek, Folke Sjöberg, Robert G. Hahn

**Affiliations:** 1 Department of Biomedical and Clinical Sciences (BKV), Linköping University, Linköping, Sweden; 2 Department of Surgery, Linköping University Hospital, Linköping, Sweden; 3 Research Unit, Södertälje Hospital, 152 86 Södertälje, Sweden; 4 Karolinska Institutet at Danderyd’s Hospital (KIDS), Stockholm, Sweden

**Keywords:** Capillary permeability, Physiology, Serum albumin, Pharmacokinetics, Therapy

## Abstract

**Background:**

We investigated whether plasma volume (PV) expansion of 20% albumin is larger when the fluid is administered rapidly compared with a slow infusion.

**Methods:**

In this open-labeled randomized interventional controlled trial, 12 volunteers (mean age, 28 years) received 3 mL/kg of 20% albumin (approximately 225 mL) over 30 min (fast) and 120 min (slow) in a cross-over fashion. Blood hemoglobin and plasma albumin were measured on 15 occasions during 6 h to estimate the PV expansion and the capillary leakage of albumin and fluid.

**Results:**

The largest PV expansion was 16.1% ± 6.5% (mean ± SD) for fast infusion and 12.8% ± 4.0% for slow infusion (*p* = 0.52). The median area under the curve for the PV expansion was 69% larger for the fast infusion during the first 2 h (*p* = 0.034), but was then similar for both infusions. The half-life of the PV expansion did not differ significantly (median, 5.6 h versus 5.4 h, *p* = 0.345), whereas the intravascular half-life of the excess albumin was 8.0 h for fast infusion and 6.3 h for slow infusion (*p* = 0.028). The measured urine output was almost three times larger than the infused volume. The plasma concentration of atrial natriuretic peptide (MR-proANP) accelerated the capillary leakage of albumin and the urine flow.

**Conclusions:**

The intravascular persistence of albumin was longer, but the fluid kinetics was the same, when 20% albumin was infused over 30 min compared with 120 min. We found no disadvantages of administering the albumin at the higher rate.

*Trial registration* EU Clinical Trials Register, EudraCT2017-003687-12, registered September 22, 2017, https://www.clinicaltrialsregister.eu/ctr-search/trial/2017-003687-12/SE

**Supplementary Information:**

The online version contains supplementary material available at 10.1186/s40635-022-00458-3.

## Background

Albumin has become the colloid fluid of choice in the intensive-care setting. Albumin has a larger and longer-lasting plasma-volume expansion effect than has been observed with crystalloid fluid [1–4]. This characteristic is determined by the osmolality and colloid osmotic pressure of the fluid, as well as by the elimination mechanisms of the body.

The rate of infusion may also be important for clinical efficacy. Statkevicius et al. recently compared 5% albumin administered over 30 min and 180 min after surgery and found the rapid infusion to be more effective [5]. The situation, however, remains unclear for 20% albumin, which exerts a pronounced volume effect [6, 7]. As 20% has a high oncotic pressure, a fast infusion might cause a more pronounced rise in plasma oncotic pressure resulting in intravascular fluid overload due to a powerful recruitment of extravascular fluid [6]. However, hormonal adjustment might counteract such overload. For example, a fast infusion might stimulate release of atrial natriuretic peptide (ANP) from the heart, which increases the capillary permeability and accelerates the urine flow [8].

The objective of the present study was to compare the plasma dilution, which mirrors the plasma volume expansion, when 20% albumin is infused at different rates in volunteers. Another objective was to investigate how much the recruitment of extravascular fluid prolongs the plasma dilution for the two infusion rates.

Our primary hypothesis was that a fast infusion increases the plasma volume more than a slow infusion. The rationale is that that elimination of 20% albumin is slow [7] and, therefore, one could anticipate a greater intravascular volume expansion when given fast. A secondary hypothesis was that recruitment of fluid from the extravascular space would markedly prolong the half-life of the plasma volume expansion. This could occur if extravascular fluid was continuously recruited rather than only during the infusion.

The study methods consisted of mass balance calculations and volume kinetic analysis of the albumin and fluid components of 20% albumin. We measured the mid-regional pro-atrial natriuretic peptide (MR-proANP) to control for the role of ANP in this setting. The results should have clinical applicability, as the kinetics of 20% albumin is quite similar in volunteers as in clinical patients with and without trauma-induced inflammation [7, 9].

## Materials and methods

This study is an open-labeled interventional randomized unblinded controlled trial with cross-over design where 12 healthy participants (six male and six female) underwent two infusion experiments, 3–20 weeks apart, where they received 3 mL/kg of 20% albumin by intravenous infusion during either 30 min (“fast infusion”) or 120 min (“slow infusion”).

The Regional Ethics Committee of Linköping (Dnr 2017/478-31) approved the study. The Swedish Medical Products Agency (Eudra-CT 2017-003687-12) also approved the protocol. The project also included a study arm with iso-oncotic albumin (clinicaltrials.gov NCT03453320), but the control groups differed due to logistical issues and the results will be presented elsewhere.

All participants gave informed consent orally and in writing. Inclusion criteria were an age between 18 and 60 years, and absence of medical disease and medication. Exclusion criteria were pregnancy, difficulties with placement of venous cannulas, and severe allergy. The study was conducted in compliance with the Declaration of Helsinki.

### Procedure

A statistician prepared 12 envelopes for randomization of the participants to start with either the slow or the fast infusion. The envelopes were opened one day prior to the first infusion by the investigators.

All participants fasted from midnight before the study and throughout the study period. However, they were allowed to drink 2 dL of liquid and eat one sandwich 1.5 h (h) prior to arrival at the research faculty at the hospital.

A venous cannula was placed in the right arm and another in the left for blood sampling and fluid infusion, respectively. The participants emptied their bladders 30 min before the study started and were then placed in a supine position until the end of the experiment. Baseline samples were withdrawn, and the participants then received the infusion. Blood was sampled on 15 occasions over a period of 6 h.

When albumin was administered over 30 min, the excreted urine was measured and sampled just before the infusion was initiated, at 30 min after the infusion ended, and at 6 h. When albumin was administered over 2 h, the excreted urine was measured and sampled just before and at the end of the infusion, and at 6 h.

### Blood and urine analyses

Whole blood was analyzed for hemoglobin (Hgb) concentration and hematocrit with a coefficient of variation (CV) of 1.0%, as given by duplicate samples at baseline, using a Cell-Dyn Sapphire instrument (Abbott Diagnostics, Abbott Park, IL, USA). Plasma was analyzed for albumin, creatinine, sodium, and potassium on a Cobas 8000 system (Roche Diagnostics, Basel, Switzerland) at the hospital’s certified central laboratory (CVs were 2.3%, 1.9%, 0.7%, and 1%, respectively, as given by the laboratory).

The plasma colloid osmotic pressure (COP) was measured in our research laboratory on an Osmomat 050 device (Gonotec, Berlin, Germany) with a CV of 2%.

The plasma concentration of the mid-regional pro-atrial natriuretic peptide (MR-proANP) at baseline and 30 min after the infusion ended was analyzed by radioimmunoassay (Brahms MR-proANP Kryptor, Henningsdorf, Germany) with a CV of < 3.5%. The manufacturer reports a median value in healthy humans of 46 pmol/L.

Urine was analyzed for creatinine on the Cobas 8000 system with a CV of 1.9%.

### Mass balance

The baseline plasma volume (PV_o_) was estimated from the height and weight of the participants, as suggested by Nadler et al. [10]. The PV change to a any later Time t of the experiment (PV_t_) was calculated based on the hemodilution curve, with correction for sampled blood volume, as described previously [6].

The albumin mass was taken as the product of the plasma volume (PV) and the plasma albumin concentration (P-Alb). Multiplication with PV is necessary because P-Alb is diluted by the infused fluid volume and by the oncotic-driven recruitment of extravascular fluid, which gives an unbalanced relationship between P-Alb and the intravascular albumin mass.

*Capillary leakage.* The net capillary leakage of albumin was obtained as the change in albumin mass, with correction for the infused amount of albumin, between baseline (time 0) and a later time t [7]. The following equation was used:

$${\text{Albumin leakage }} = {\text{infused albumin }} + \, \left( {{\text{PV}}_{{{\rm o}}} \times {\text{P-Alb}}_{{{\rm o}}} } \right) {-} \left( {{\text{PV}}_{{{\rm t}}} \times {\text{P-Alb}}_{{{\rm t}}} } \right).$$

*Half-life.* The half-life of the infused albumin was obtained from the logarithm of the slope of the albumin mass, given as $\left[ {\left( {{1 } + {\text{PV}}_{{{{\rm dil}}}} } \right)_{{}} {\text{P-Alb}}_{{{{\rm t}} }} {-}{\text{P-Alb}}_{{{\rm o}}} } \right]$ versus time, when an apparent first-order elimination had been established post-infusion [7]. For each experiment, the half-life of the decay of the PV expansion was estimated in the same way for the albumin mass.

### Albumin and fluid kinetics

A one-compartment model was used to study the kinetics of the infused *albumin mass* throughout the entire experiment. In this model, albumin mixed in fluid was infused at a rate *R*_o_ into a central body fluid space *V*_c_, which was then expanded to *v*_c_. The net capillary leakage was given by the rate constant *k*_b_ (“net” denotes that *k*_b_ represents the true capillary leakage minus the albumin added via the lymph). The dependent variable was the product of the increase in measured P-Alb and the plasma dilution; the latter was given by ((Hgb_o_/Hgb_t_) − 1)/(1 − hematocrit_o_), where the subscript o denotes the baseline and *t* a later time. Minor correction of the plasma dilution for blood sampling was made [6].

The kinetics of the infused *fluid volume* was evaluated using a model with micro-constants that is developed for studies of 20% albumin [11]. This model has one infusion, one absorption route, and two elimination routes and was fitted to the plasma dilution and the urine output, which served as the dependent variables.

Absorption occurred from an extravascular source, which is likely to be the interstitial fluid space, by (supposedly) oncotic forces and at a rate that was determined by a constant denoted *k*_21_. The interstitial fluid volume at baseline (ICF_o_) was assumed to contain fluid accounting for 15% of the body weight [12].

Fluid volume was eliminated by urine output (*k*_10_) and capillary leakage (*k*_b_).

This “base model” was expressed by the following differential equations:

$${\text{d}}v_{{{\rm c}}} /{\text{dt}} = {\text{ R}}_{{{\rm o}}} {-}k_{{{\rm b}}} \left( {v_{{{\rm c}}} {-}V_{{{\rm c}}} } \right){-}k_{{{1}0}} \left( {v_{{{\rm c}}} {-}V_{{{\rm c}}} } \right) \, + k_{{{21}}} {\text{ICF}}_{{{\rm o}}} ,$$

$${\text{dICF}}/{\text{dt }} = \, {-}k_{{{21}}} {\text{ICF}}_{{{\rm o}}} ; {\text{d}}U/{\text{dt }} = k_{{{1}0}} (v_{{{\rm c}}} {-}V_{{{\rm c}}} ),$$

where *V*_c_ is the baseline and *v*_c_ the expanded central volumes, and *U* the measured urine output. The rate parameter *k*_21_ does not come into play before the infusion begins.

The fixed parameters in the *albumin model* (*V*_c_ and *k*_b_ for albumin) were estimated simultaneously for all 24 experiments using the first-order conditional estimation extended least-squares (FOCE ELS) search routine in the Phoenix software for nonlinear mixed effects (NLME), version 8.2 (Pharsight, St. Louis, MO) and the additive model for the within-subject variability. The dependent variable was P-Alb corrected for plasma dilution.

The fixed parameters in the *fluid model* (*V*_c_, *k*_10_, *k*_b_, and *k*_21_) were estimated in the same way. Here, the dependent variables were the frequently measured plasma dilution and the urine output measured at 1 h and 6 h.

Both base models were both refined by adding individual-specific *covariates.* Nine potential covariates were examined. Age, body weight, gender, Hgb_o_, and urine osmolality and urine creatinine at baseline were entered once for each patient. Plasma creatinine, and MR-proANP were measured twice per experiment and applied at the point in time when measured. The change in plasma albumin from baseline was entered as a time-varying covariate 15 times per experiment (at the same time points as Hgb was measured).

### Outcome measures

The primary outcome measure was the plasma dilution, which equals the relative change in plasma volume, at 6 h and at the end of the infusions of hyper-oncotic albumin. Secondary outcome measure was the intravascular half-life of the excess amount of albumin and the plasma dilution as obtained by mass balance and kinetic analysis.

### Statistics

Power analysis prior to the study was based on the previously obtained mean ± standard deviation (SD) values of 15.8% ± 4.9% for the plasma volume expansion at end of infusing 3 mL/kg of 20% albumin [11]. We aimed at identifying a difference in plasma volume expansion of 20% at the *p* < 0.05 level and with a certainty of 80%. This calculation yielded 22 experiments.

The measured variables were reported as the mean ± SD and, when appropriate, as the median and interquartile range (IQR). Differences between the two infusions were studied, depending on the distribution of the data, by the paired *t* test or Wilcoxon’s matched-pair test. *p* < 0.05 was deemed statistically significant.

The excess amount of intravascular albumin and the plasma volume expansion over time was expressed as the area under the curve (AUC) which was calculated by the linear trapezoid method.

Kinetic parameters were reported as the best estimate and 95% confidence interval (CI). A new parameter (fixed or covariate) was accepted if its 95% CI did not include 0 and the inclusion decreased the − 2 log likelihood (− 2 LL) for the model by > 3.8 points (*p* < 0.05).

## Results

**Fig. 1:**
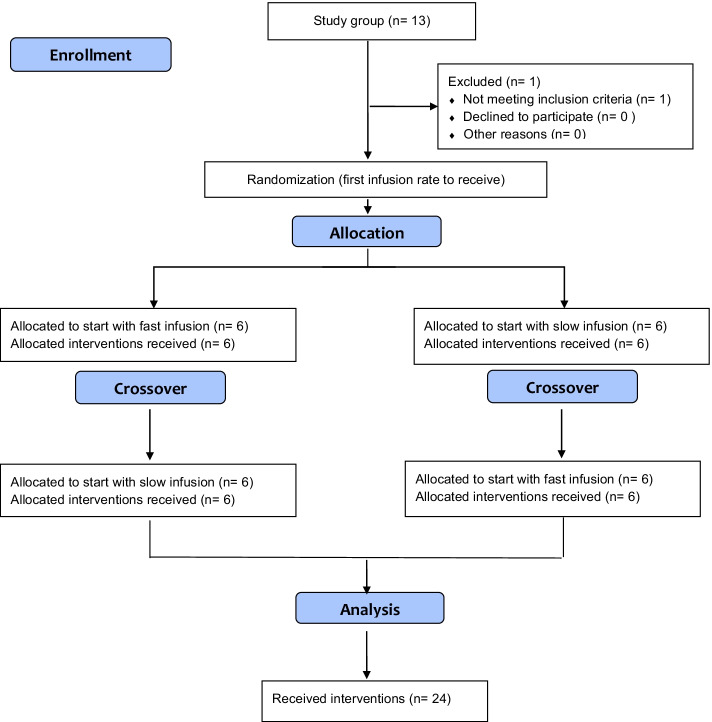
CONSORT flow diagram of the volunteer enrollment

The participants were studied between February 2018 and November 2018. One participant was excluded due to difficulties with the placement of the two venous cannulas before the infusion was administered. This subject was replaced by another volunteer. The finally included volunteers were 28 ± 10 years old, had a body weight of 75 ± 10 kg, and body mass index of 24.2 ± 2.8 kg/m^2^. A CONSORT flow diagram is shown in Fig. 1.

The volume of infused 20% albumin amounted to 225 ± 31 mL.

**Fig. 2:**
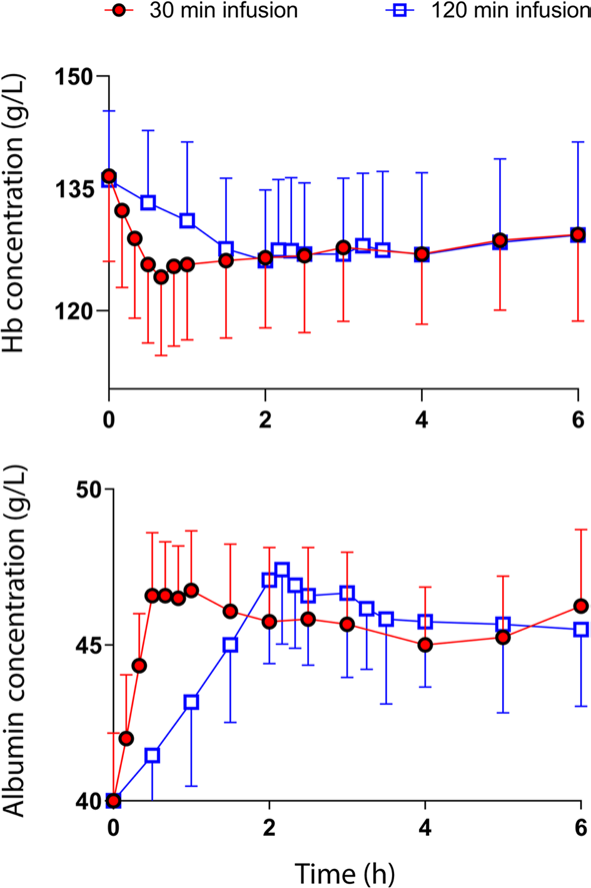
Raw data. **A** The blood hemoglobin and **B** plasma albumin concentration during and after infusion of 3 ml/kg of 20% albumin over 30 min and 120 min in volunteers. Data are the mean (SD)

The measured blood Hgb and plasma albumin concentrations are shown in Fig. 2 and the results of other measurements and calculations in Table 1.

**Table 1:** Demographic and clinical characteristics of the participants in the 20% albumin infusion study

Variable	Fast infusion	Slow infusion	*p*-value
Plasma volume expansion (mL/kg)			
0.5 h	5.4 ± 2.2	1.5 ± 1.6	0.001
2 h	4.2 ± 2.6	4.9 ± 1.6	0.46
6 h	2.5 ± 2.4	2.5 ± 2.5	0.92
AUC excess albumin (g min/kg)			
0–2 h	50.3 (40.5–58.2)	28.2 (24.8–31.8)	0.003
Half-life albumin mass (h)	8.0 (5.4–11.6)	6.3 (4.4–8.4)	0.028
AUC ∆plasma volume (L min/kg)			
0–2 h	0.44 (0.40–0.65)	0.26 (0.19–0.42)	0.034
0–6 h	1.08 (0.73–1.90)	0.97 (0.67–1.68)	0.31
Half-life plasma volume (h)	5.6 (3.4–7.7)	5.4 (2.0–10.1)	0.347
Colloid osmotic pressure (mmHg), 0 min	25.0 ± 1.0	25.1 ± 1.4	0.85
Recruited extravascular fluid (mL/mL)^1^	3.1 ± 1.3	3.1 ± 1.4	0.84
Mean arterial pressure (mmHg)			
0 h	92 ± 11	93 ± 10	0.71
1 h	90 ± 15	91 ± 8	0.73
2.5 h	90 ± 12	91 ± 11	0.71
6 h	94 ± 13	94 ± 10	0.96
Thrombocytes (10^9^/L)			
0 h	201 ± 50	190 ± 37	0.10
6 h	193 ± 46	182 ± 41	0.02
Plasma sodium (mmol/L)			
0 h	141.3 ± 2.0	141.0 ± 1.6	0.61
6 h	142.1 ± 1.7	141.6 ± 1.8	0.36
Plasma potassium (mmol/L)			
0 h	3.9 ± 0.3	3.9 ± 0.2	0.68
6 h	3.9 ± 0.2	3.9 ± 0.2	0.55
Serum creatinine (µmol/L)			
0 h	85 ± 14	85 ± 14	0.94
6 h	78 ± 13	79 ± 14	0.54
Urinary creatinine (mmol/L)			
0 h	15.1 ± 6.3	14.0 ± 4.5	0.30
6 h	9.5 ± 5.9	9.9 ± 4.4	0.82

Data are the mean ± SD or median (interquartile range)

AUC = area under the curve. ^**1**^ for this calculation, see reference [6]

The mean (SD) MR-proANP was 43 ± 12 and 44 ± 17 pmol/L, respectively, before the fast and slow infusions were started. Thirty minutes after they ended, these mean (SD) concentrations had increased to 54 ± 24 and 55 ± 24 pmol/L, respectively. The change was significant by *p* < 0.01.

### Mass balance

**Fig. 3:**
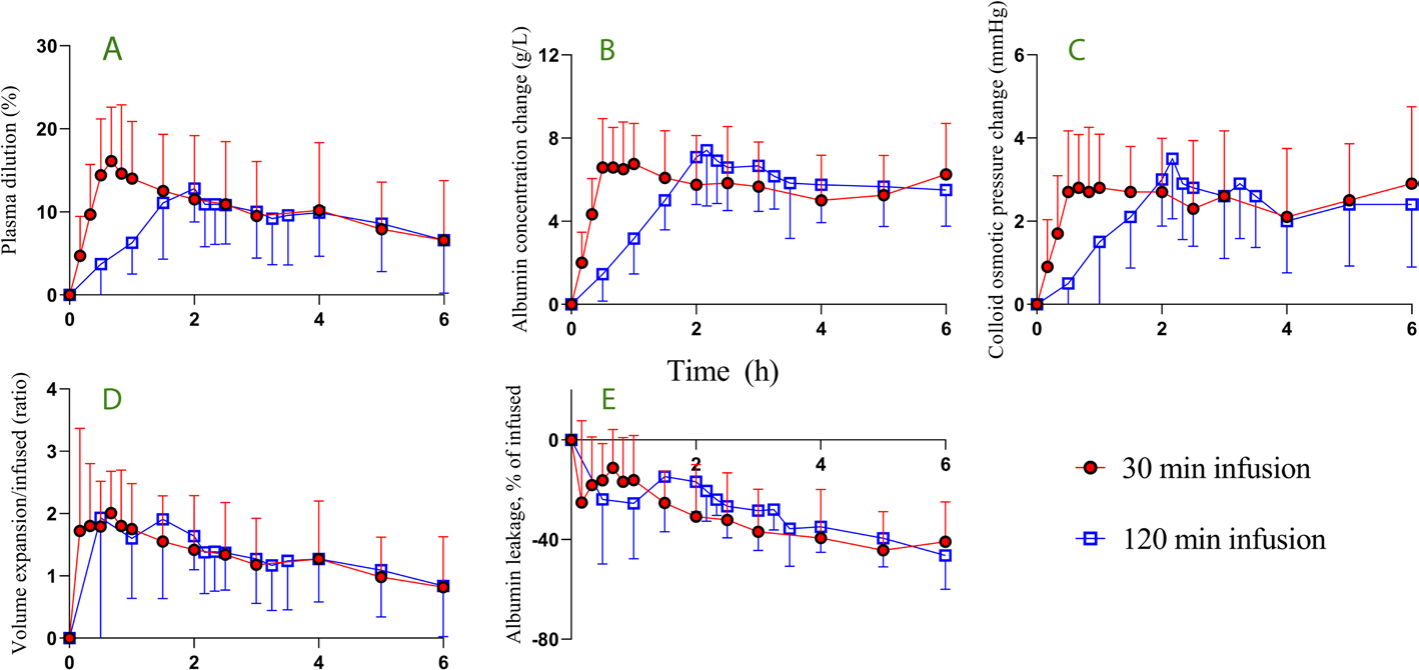
Mass balance. **A** Plasma dilution. **B** Changes in plasma albumin concentration. **C** Changes in plasma colloid osmotic pressure. **D** Plasma volume expansion divided by the infused volume of 20% albumin. **D** Capillary leakage of albumin expressed as a percentage of the infused amount of albumin. All data are the mean (SD)

The largest mean (SD) plasma volume expansion during the fast infusion experiment was 16.1% ± 6.5% and occurred 10 min after the infusion ended. The corresponding maximum value for the slow infusion was 12.8% ± 4.0 (*p* = 0.52) and recorded at the end of the infusion (Fig. 3A). The plasma volume expansion at 6 h was 4.5 (IQR 2.0–8.4)% after the fast infusion and 5.2 (1.4–12.6)% after the slow infusion (*p* = 1.0).

The plasma albumin concentration was 40.0 ± 2.2 g/L (mean ± SD) at baseline prior to the fast infusion and 40.0 ± 2.0 g/L prior to the slow infusion. These concentrations had increased by 6.6 ± 2.3 g/L and 7.1 ± 2.3 g/L, respectively, at the end of the infusions (*p* = 0.62; Fig. 3B).

The plasma COP was 25.0 ± 1.2 prior to the fast infusion and 25.1 ± 1.4 prior to the slow infusion. The increase in COP was 2.7 ± 1.5 mmHg at the end of the fast infusion and 3.0 ± 1.1 mmHg after the slow infusion (*p* = 0.65; Fig. 3C).

At the end of infusion, the fast infusion had increased the plasma volume by twice the administered fluid volume, while the slow infusion had expanded the plasma volume by 1.6 times the infused volume (*p* = 0.19; Fig. 3D).

At 6 h, the net capillary leakage of albumin amounted to 41% ± 16% of the infused amount after the fast infusion and 46% ± 14% after the slow infusion (*p* = 0.53; Fig. 3E).

The intravascular half-life of albumin post-infusion was longer when 20% albumin was infused fast compared to the slow infusion 8.0 (5.4–11.6) h versus 6.3 (4.4–8.4) h (*p* = 0.028). However, the half-life of the plasma volume expansion did not differ significantly, being 5.6 (3.4–7.7) h versus 5.4 (2.0–10.1) h (*p* = 0.347).

The median AUC for the excess amount of albumin during the first 2 h of the experiments was 78% larger for the fast infusion (*p* < 0.003) and the plasma volume expansion was 69% larger for the fast infusion as compared to the slow infusion (*p* < 0.034; Table 1). After the first 2 h the plasma volume curves were virtually identical, while plasma albumin was slightly more increased after the slow infusion (Fig. 3A, B). The AUC for the plasma volume expansion during the entire experiment still did not differ significantly (Table 1).

The cumulative urinary output at 6 h amounted to 631 ± 354 mL and 612 ± 242 after the fast and slow infusion experiments, respectively (*p* = 0.83), which represented 2.9 ± 1.6 and 2.8 ± 1.2 times the infused volume.

### Kinetic analyses

**Fig. 4:**
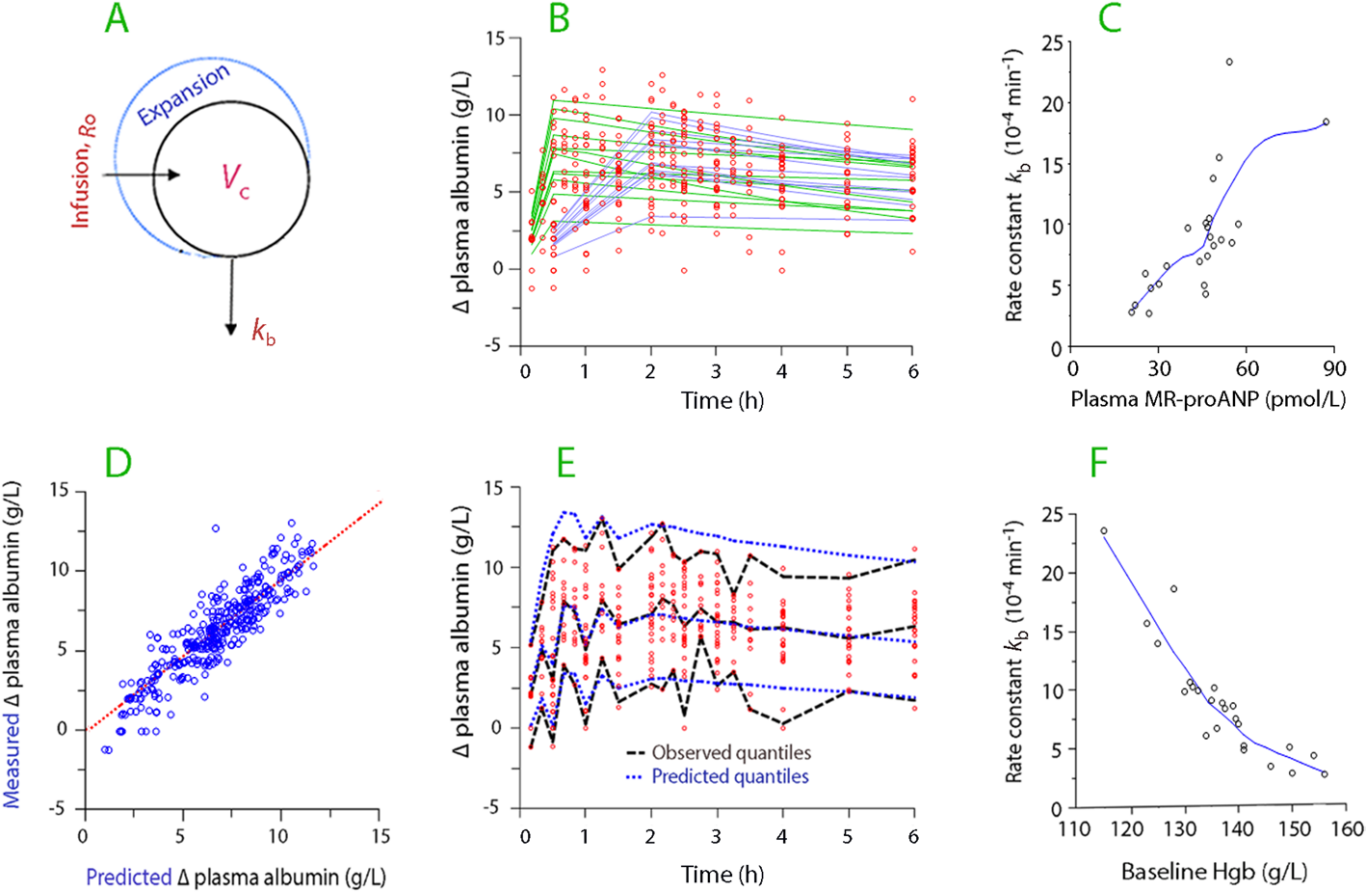
Albumin kinetics. **A** Schematic drawing of the volume kinetic model. **B** Curve-fit in the final model for the dilution-corrected change in plasma albumin concentration versus time. **C** Increase in the rate constant for capillary leakage of albumin for increasing plasma MR-proANP concentrations. **D** Predicted versus measured dilution-corrected change in plasma albumin concentration in the final model. **E** Original data (red points) with the associated 5%, 50%, and 95% quantiles (black lines) and the corresponding quantiles obtained by predictive check based on 1000 simulations using the model parameters in the final model (blue lines). A small difference between observed and predicted quantiles indicates good model performance. **F** Inhibitory effect of high blood Hgb concentrations on the capillary leakage of albumin.

Relevant graphic output of the analysis of the kinetics of the *infused albumin* is shown in Fig. 4 (for underlying data, Table 2, top).

**Table 2:** Population kinetic parameters for infused albumin mass in the final model

Kinetic parameter	Covariate	Covariate model	Best estimate	95% CI	CV%	− 2 LL
**Albumin kinetics**						
tv*V*_c_ (L)			5.89	5.16–6.62	6.3	
tv*k*_10_ (10^–4^ min^−4^)			8.65	5.86–11.4	16.4	1213
* k*_10_	Hgb baseline	Power	− 5.50	− 6.96 to − 4.04	− 13.5	1203
* V*_c_	Albumin baseline	Power	4.32	1.60–7.03	31.9	1193
* k*_10_	120-min infusion	Exponential	0.40	0.02–0.78	47.8	1187
**Fluid kinetics**						
tv*V*c (L)			2.09	1.45–2.73	15.6	
tv*k*_10_ (10^–3^ min^−1^)	–		11.5	8.4–14.5	13.4	
tv*k*_21_ (10^–4^ min^−1^)	–		3.19	1.49–4.88	27.1	− 528
tv*k*_b_ (10^–3^ min^−1^)			12.5	3.90–21.0	35.0	− 582
* k*_21_	∆ P-Albumin	Linear	0.083	0.057–0.108	16.0	− 609
* k*_10_	MR-proANP	Power	0.80	0.37–1.23	28.7	− 624
* k*_10_	U-creatinine, baseline	Exponential	− 0.96	− 1.80 to − 0.11	− 45.0	− 632

Shown are the typical values (tv) for the fixed parameters in the group, followed by individual-specific covariates

tv = typical value for the group. CI = confidence interval. CV% = coefficient of variation (inter-individual)

Mean blood Hgb at baseline 137 g/L and mean plasma albumin 40.0 g/L

LL = log likelihood for the model during development. Decrease by > 3.8 points = *P* < 0.05

Mean MR-proBNP = 49 pmol/L and urine creatinine 10.3 mmol/L

Covariance analysis showed that the net capillary leakage of albumin, as represented by the rate constant *k*_b_, was accelerated by plasma MR-proANP in the high range (Fig. 4C) and by low Hgb at baseline (Fig. 4F).

**Fig. 5:**
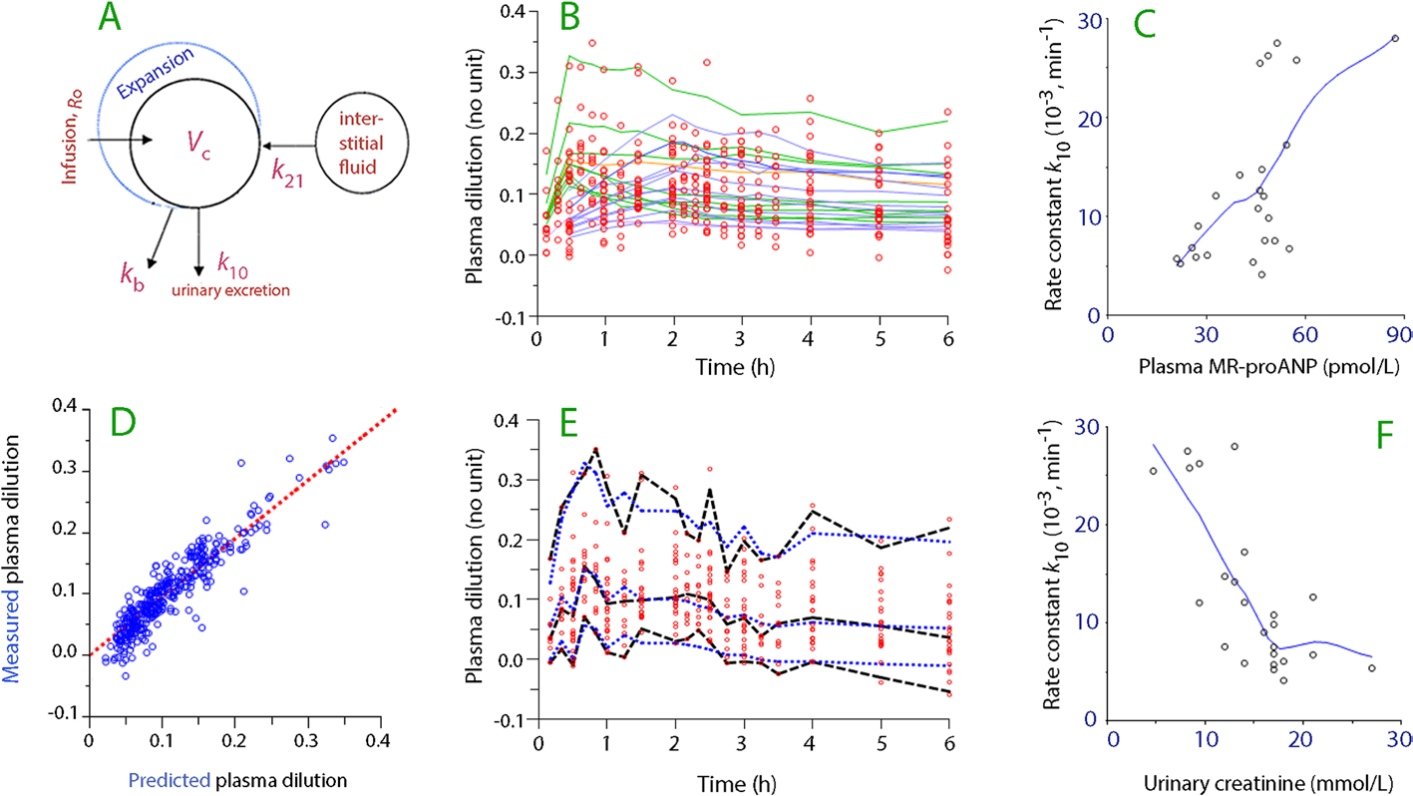
Fluid volume kinetics. **A** Schematic drawing of the volume kinetic model. **B** Curve-fit in the final model for the plasma dilution versus time. **C** Correlation between the plasma MR-proANP concentration and the rate constant for urine flow (*k*_10_). **D** Predicted versus measured plasma dilution in the final model. **E** Measured plasma dilution (red points) with the associated 5%, 50%, and 95% quantiles (black lines) and the corresponding quantiles obtained by a predictive check based on 1000 simulations using the model parameters in the final model (blue lines). **F** Inverse correlation between the urinary creatinine concentration at baseline and the rate constant for urine flow (*k*_10_)

Graphic output from the analysis of *fluid kinetics* is given in Fig. 5 (for underlying data, Table 2, bottom). The rate constant for urinary excretion of intravascular fluid (*k*_10_) indicated a half-life of the plasma volume expansion of 60 min. The half-life of the plasma volume expansion would be reduced to 28 min if we consider the combined effects of urinary flow and capillary leakage of fluid [calculated as ln 2/(*k*_10_ + *k*_b_)]. However, the continuous recruitment of fluid from the extravascular space (*k*_21_) was powerful enough to prolong the half-life of the plasma volume expansion to the 5 h that was obtained by mass balance.

The covariance analysis confirmed that the intensity of the fluid recruitment by *k*_21_ was directly dependent on the elevation of plasma albumin from baseline that resulted from the infusion of 20% albumin. The covariance analysis also showed that the urine flow was accelerated by a high plasma MR-proANP concentration (Fig. 5C) and a low urinary creatinine concentration at baseline (Fig. 5F).

**Fig. 6:**
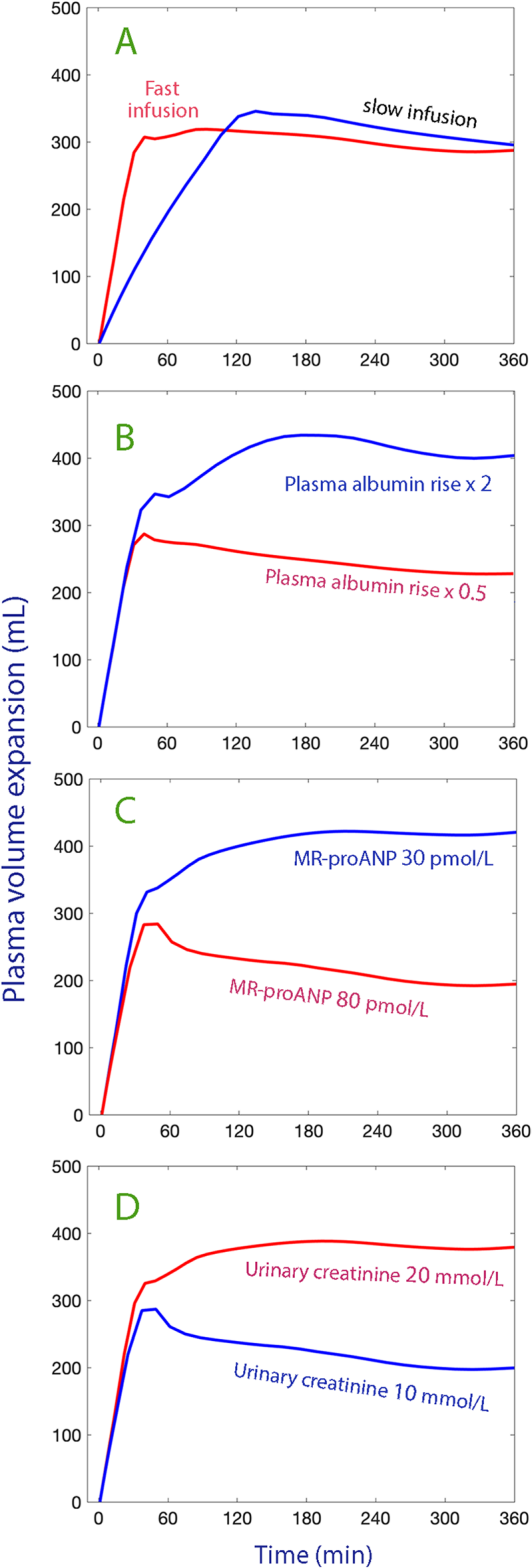
Factors of importance to the plasma volume expansion. Computer simulations contrasting **A** rates of infusion **B** excessive (× 2) or reduced (× 0.5) increase in plasma albumin relative to the measured concentrations **C** the plasma MR-proANP concentration was high or low **D** whether the baseline urinary concentration of creatinine was high or low. The data from Table 2 were used. Simulations were performed by setting all kinetic parameters to the mean value except for the parameter that was varied. Subplots **B**–**D** were based on the kinetic data from all 24 experiments, but the plasma albumin measured during the 30-min infusion only

The influence of the covariates on the plasma volume expansion is illustrated by simulations in Fig. 6.

## Discussion

### Key results

The results show that 20% albumin was an effective plasma volume expander regardless of infusion rate. The fast infusion expanded the plasma volume much more at 30 min, but only 25% of the slow infusion had been administered at that time. The area under the curve for the plasma volume expansion during the first 2 h was still 65% larger for the fast infusion and was not compensated by a less pronounced long-term expansion (primary hypothesis), which would else be expected according to pharmacokinetic theory. Hence, at 6 h the plasma volume was virtually identical for the two infusion rates. As much as one third of the maximum volume expansion remained in the intravascular space at 6 h.

The half-life of the intravascular persistence of the infused fluid volume was prolonged from 0.5 h to 5 h by gradual recruitment of extravascular fluid due to the rise in plasma albumin, which is apparently an important characteristic of the clinical efficacy of 20% albumin (secondary hypothesis).

Capillary leakage of albumin occurred continuously throughout the study, but more slowly when the infusion was given fast. The fluid volume kinetics did not differ between the two infusion rates, but it appeared to be more strongly governed by individual-specific physiological factors (covariates) than the capillary leakage of albumin.

### Diuresis and MR-proANP

The marked reduction of urinary creatinine during the experiment (Table 1) illustrates that 20% albumin is a diuretic. The volume of excreted urine was almost three times larger than the infused fluid volume.

The urine output was affected by two individual-specific factors. The first factor was the urinary creatinine concentration, which is high when the kidneys concentrate the urine to retain water (Fig. 6D). We have observed the same retarding effect of urinary creatinine on the excretion of infused crystalloid fluid [13]. High urinary creatinine is caused either by acute dehydration or a low habitual intake of water [14]. The latter possibility should be at hand in the present study as the subjects were healthy volunteers.

The second factor was the plasma MR-proANP concentration (Fig. 6C). Prior to this study, we had concerns that the rapid infusion of 20% albumin would increase the plasma volume sufficiently to markedly elevate the MR-proANP concentration, which is a precursor of ANP that is excreted from the atrium of the heart in response to distention (increased cardiac preload). The increase reached only 25% in both groups, but the between-patient variation in MR-proANP was still large enough to render this variable a statistically significant predictor of the urine flow.

Statkevicius et al. recently compared slow and rapid infusions of 5% albumin after abdominal surgery and reported a greater increase in MR-proANP for a 30 min infusion than for a 180 min infusion. However, they found no difference in the transcapillary escape rate of albumin [5].

The plasma concentration of ANP doubles in response to rapid infusion of a crystalloid fluid [15, 16]. The urine flow increases also in response to modest elevations of brain natriuretic peptide, which is closely related to ANP [17]. This hormone is assessed clinically by quantifying its proform, NT-proBNP, which is the chief biomarker of heart failure. In septic patients 20% albumin caused a greater early rise in NT-proBNP than crystalloid fluid, which was associated with better prognosis in the absence of shock but poorer prognosis in the presence of shock [18]. In conscious healthy subjects the brain natriuretic peptide was doubled in response to crystalloid volume loading, but changes were negligible when crystalloid loading was performed during general anesthesia [19, 20].

### Half-life of albumin

Our finding that rapid infusion prolongs the intravascular half-life of albumin receives indirect support from other authors. Margarson and Soni infused 200 mL 20% albumin in volunteers over only 2 min [21]. After 4 h, as much as 79% of the infused albumin mass remained in the blood, which compares to the 60% measured in the present study (Fig. 3E). In septic patients, they reported that 69% of the administered albumin mass remained in the bloodstream after 4 h [21], which is still a greater intravascular retention than in our volunteers. By contrast, von Seth et al. [22] studied the capillary leakage of albumin in septic pigs and found no difference between fast and slow infusions.

There are three possible explanations for the longer intravascular half-life of albumin when 20% albumin is administered rapidly:

1. Rapid and powerful transcapillary recruitment of extravascular fluid concentrates the interstitial albumin which, based on animal experiments [23, 24], slows down the albumin leakage.
2. The recruited fluid might partially or entirely consist of lymph, which contains 40% as much albumin per volume as does plasma [25]. This inflow of albumin would artificially prolong the intravascular half-life as measured in the present study.
3. Very rapid increase of the plasma albumin concentration could partially plug the endothelial intercellular junctions and thereby limiting capillary leakage. This mechanism has been suggested by laboratory experiments, but was not confirmed in septic patients [26].

Explanation 2 receives support from the kinetic analysis, where *k*_b_ is much lower for albumin than for fluid. Explanations 1 and 3 are supported by the absence of net capillary leakage of albumin during the last part of the infusions (Fig. 3E). The changed direction of the net capillary leakage at this time might be due to that lymphatic return of albumin outweighed a depressed capillary leakage of albumin.

The longer intravascular persistence of infused albumin was not reflected in a larger plasma volume expansion. This might be due to that fluid equilibrates faster and more easily between body fluid compartments than albumin does.

### Limitations

The diuretic effect of 20% albumin reported in the present study should not be uncritically extrapolated to severely ill patients. The SWIPE Trial compared 20% and 5% albumin in the intensive-care setting and found no difference in urine output; however, more fluid volume was given to the 5% albumin group [27]. The ALBIOS Trial compared septic patients randomized to 20% albumin and crystalloids; here, too, was the urine output the same, but a larger fluid volume was administered to those in the crystalloid group [28].

We included only volunteers who had a normal plasma albumin concentration (mean 40 g/L) before the infusions started. However, we have previously observed that the kinetics of 20% albumin is no different in healthy volunteers than it is in postoperative and post-burn patients, although these clinical groups had mean plasma albumin being 60% of the concentrations reported here [7, 9].

The measurements taken just after voiding during the study (at 75 and 165 min) were deleted because the efforts then made by the volunteers concentrated the blood.

Our kinetic models visualize changes in flow rates between body compartments, whereas the baseline flows are not included.

The kinetic model for albumin was relatively simple, but was based on the entire concentration–time curve corrected for dilution. However, the first 60–90 min after the infusions ended could show fluctuating values, even to the extent that a “steady state” developed. The balance between body fluid compartments was apparently not yet in order, perhaps due to recruitment of lymph. We preferred to report the apparent intravascular half-life of albumin based on mass balance applied to the terminal part of the experiment. This approach yielded values that better represented most elimination curves.

Statistical adjustment for multiple comparisons was not applied since our key outcome measures were few (plasma volume expansion at the end of infusion and at 6 h and the half-lives of the plasma volume expansion and excess albumin). We cannot rule out that the longer half-life of the excess amount of albumin for the fast infusion was due to a random significance, but the risk does not seem to be great.

We believe that the generalizability of the present study is high, as previous studies using the same protocol show that the kinetics of 20% albumin is quite stable between different clinical situations and similar to what is found in volunteers [7, 9, 11].

## Conclusion

Infusion of 20% albumin over 30 and 120 min yielded stable plasma volume expansion curves. The expansion was larger for the fast infusion during the first 2 h, but quite similar for the two series of experiments between 2 h and 6 h. Calculations showed that the intravascular persistence of albumin was longer for the 30-min infusion than for the 120-min infusion while the fluid kinetics did not differ. There was no difference in MR-proANP response between the two infusions, but inter-individual differences in this hormone affected the capillary leakage of albumin and the urinary output. Overall, we found no negative effects from the fast administration.

## Supplementary Information

**Additional file 1.** Supplemental File: All original data for the study "Fast versus slow infusion of 20% albumin: a randomized controlled cross-over trial in volunteers".

## Data Availability

All data are available as Additional file 1.

## Abbreviations

ANP: Atrial natriuretic peptide
CV: Coefficient of variation
COP: Coefficient of variation
dL: Deciliter
FOCE ELS: First-order conditional estimation extended least-squares
h: Hours
Hgb: Hemoglobin
ICF: Extravascular (probably interstitial) fluid
IQR: Interquartile range
*k*_10_: Rate constant for urinary output
*k*_21_: Rate constant for absorption of extravascular fluid to the plasma
min: Minutes
MR-proANP: Mid-regional pro-atrial natriuretic peptide
P-Alb: Plasma albumin
PV_o_: Plasma volume at baseline
PV_t_: Plasma volume at time t of the experiment
R_o_: Infusion rate
*k*_b_: Rate constant for capillary leakage
*V*_c_: Conversion factor between mass and concentration (albumin) or between volume and plasma dilution (fluid kinetics)
*v*_c_: Expanded volume of *V*_c_
SD: Standard deviation

## References

[1] Jacob M, Chappell D, Conzen P, Wilkes MM, Becker BF, Rehm M (2008). Small-volume resuscitation with hyperoncotic albumin: a systematic review of randomized clinical trials. Crit Care.

[2] Hahn RG, Lyons G (2016). The half-life of infusion fluids: an educational review. Eur J Anesthesiol.

[3] Hahn RG (2020). Understanding volume kinetics. Acta Anaesthesiol Scand.

[4] Hedin A, Hahn RG (2005). Volume expansion and plasma protein clearance during intravenous infusion of 5% albumin and autologous plasma. Clin Sci.

[5] Statkevicius S, Bonnevier J, Fisher J, Bark BP, Larsson E, Öberg CM, Kannisto P, Tingstedt D, Bentzer P (2019). Albumin infusion rate and plasma volume expansion: a randomized clinical trial in postoperative patients after major surgery. Crit Care.

[6] Zdolsek M, Hahn RG, Zdolsek JH (2018). Recruitment of extravascular fluid by hyperoncotic albumin. Acta Anaesthesiol Scand.

[7] Hasselgren E, Zdolsek M, Zdolsek JH, Björne H, Krizhanovskii C, Ntika S, Hahn RG (2019). Long intravascular persistence of 20% albumin in postoperative patients. Anesth Analg.

[8] Chappell D, Bruegger D, Potzel J, Jacob M, Brettner F, Vogeser M, Conzen P, Becker BF, Rehm M (2014). Hypervolaemia increases release of atrial natriuretic peptide and shedding of the endothelial glycocalyx. Crit Care.

[9] Zdolsek M, Hahn RG, Sjöberg F, Zdolsek JH (2020). Plasma volume expansion and capillary leakage of 20% albumin in burned patients and volunteers. Crit Care.

[10] Nadler SB, Hidalgo JU, Bloch UT (1962). Prediction of blood volume in normal human adults. Surgery.

[11] Hahn RG, Zdolsek M, Hasselgren E, Zdolsek J, Björne H (2019). Fluid volume kinetics of 20% albumin. Br J Clin Pharmacol.

[12] Guyton AC, Hall JE (1996). Textbook of medical physiology.

[13] Hahn RG (2021). Renal water conservation and the volume kinetics of fluid-induced diuresis; a retrospective analysis of two cohorts of elderly men. Clin Exp Pharm Physiol.

[14] Hahn RG (2021). Effects of diet, habitual water intake and increased hydration on body fluid volumes and urinary analysis of renal fluid retention in healthy volunteers. Eur J Nutr.

[15] Kamp-Jensen M, Olesen KL, Bach V, Schütten HJ, Engqvist A (1990). Changes in serum electrolyte and atrial natriuretic peptide concentrations, acid-base and haemodynamic status after rapid infusion of isotonic and Ringer lactate solution in healthy volunteers. Br J Anaesth.

[16] Hahn R, Stalberg H, Carlström K, Hjelmqvist H, Ullman J, Rundgren M (1994). Plasma atrial natriuretic peptide concentration and renin activity during overhydration with 1.5% glycine solution in conscious sheep. Prostate.

[17] Hahn RG, Nemme J (2020). Volume kinetic analysis of fluid retention after induction of general anaesthesia. BMC Anaesthesiol.

[18] Masson S, Caironi P, Fanizza C, Carrer S, Caricato A, Fassini P, Vago T, Romero M, Tognoni G, Gattinoni L, Latini R, Albumin Italian Outcome Sepsis Study Investigators (2016). Sequential N-terminal pro-B-type natriuretic peptide and high-sensitivity cardiac troponin measurements during albumin replacement in patients with severe sepsis or septic shock. Crit Care Med.

[19] Norberg Å, Hahn RG, Li H, Olsson J, Prough DS, Börsheim E, Wolf S, Minton R, Svensén CH (2007). Population volume kinetics predicts retention of 0.9% saline infused in awake and isoflurane-anesthetized volunteers. Anesthesiology.

[20] Nemme J, Krizhanovskii C, Ntika S, Sabelnikovs O, Vanags I, Hahn RG (2020). Hypervolaemia does not cause degradation of the endothelial glycocalyx layer during open hysterectomy performed under sevoflurane or propofol anaesthesia. Acta Anaesthesiol Scand.

[21] Margarson MP, Soni NC (2004). Changes in serum albumin concentration and volume expanding effects following a bolus of albumin 20% in septic patients. Br J Anesth.

[22] von Seth M, Lipcsey M, Engström P, Larsson A, Hillered L, Maripuu E, Widström C, Sjölin J (2017). Rapid bolus administration does not increase the extravasation rate of albumin: a randomized controlled trial in the endotoxemic pig. Shock.

[23] Renkin EM, Tucker V, Rew K, O’Loughlin D, Wong M, Sibley L (1992). Plasma volume expansion with colloids increases blood-tissue albumin transport. Am J Physiol.

[24] Bent-Hansen L (1991). Whole body capillary exchange of albumin. Acta Physiol Scand suppl.

[25] Rutili G, Arfors K-E (1977). Protein concentration in interstitial and lymphatic fluids from the subcutaneous tissue. Acta Physiol Scand.

[26] Margarson MP, Soni NC (2002). Effects of albumin supplementation on microvascular permeability in septic patients. J Appl Physiol.

[27] Mårtensson J, Bihari S, Bannard-Smith J, Glassford NJ, Lloyd-Donald P, Cioccari L, Luethi N, Tanaka A, Crisman M, Rey de Castro N, Ottochian M, Huang A, Cronhjort M, Bersten AD, Prakash S, Bailey M, Eastwood GM, Bellomo R (2018). Small volume resuscitation with 20% albumin in intensive care: physiological effects: the SWIPE randomised clinical trial. Intensive Care Med.

[28] Caironi P, Tognoni G, Masson S, Fumagalli R, Pesenti A, Romero M, Fanizza C, Caspani L, Faenza S, Grasselli G, Iapichino G, Antonelli M, Parrini V, Fiore G, Latini R, Gattinoni L, ALBIOS Study Investigators (2014). Albumin replacement in patients with severe sepsis or septic shock. N Engl J Med.

